# SOX11, SOX10 and MITF Gene Interaction: A Possible Diagnostic Tool in Malignant Melanoma

**DOI:** 10.3390/life11040281

**Published:** 2021-03-27

**Authors:** Marius-Alexandru Beleaua, Ioan Jung, Cornelia Braicu, Doina Milutin, Simona Gurzu

**Affiliations:** 1 Department of Pathology, Clinical County Emergency Hospital, Targu-Mures, Romania, 540139 Targu Mures, Romania; marius.beleaua@umfst.ro (M.-A.B.); doina.milutin@spitalmures.ro (D.M.); 2 Department of Pathology, George Emil Palade University of Medicine, Pharmacy, Sciences and Technology, 38 Gheorghe Marinescu Street, 540139 Targu Mures, Romania; jungjanos@studium.ro; 3 Research Center for Functional Genomics, Biomedicine and Translational Medicine, Iuliu Hatieganu University of Medicine and Pharmacy, 400337 Cluj-Napoca, Romania; cornelia.braicu@umfcluj.ro; 4 Research Center (CCAMF), George Emil Palade University of Medicine, Pharmacy, Sciences and Technology, 540139 Targu Mures, Romania

**Keywords:** melanoma cocktail, immunohistochemistry, SOX11, SOX10, MITF, survival

## Abstract

Malignant melanoma (MM) is a highly heterogenic tumor whose histological diagnosis might be difficult. This study aimed to investigate the diagnostic and prognostic utility of the conventional pan-melanoma cocktail members (HMB-45, melan-A and tyrosinase), in conjunction with SOX10 and SOX11 immunohistochemical (IHC) expression. In 105 consecutive cases of MMs and 44 of naevi, the IHC examination was performed using the five-abovementioned markers, along with microphthalmia transcription factor (MITF), S100, and Ki67. Correlation with the clinicopathological factors and a long-term follow-up was also done. Survival analysis was performed with Kaplan–Meier curves and compared with TCGA public datasets. None of the 44 naevi expressed SOX11, but its positivity was seen in 52 MMs (49.52%), being directly correlated with lymphovascular invasion, the Ki67 index, and SOX10 expression. HMB-45, SOX10, and tyrosinase, but not melan-A, proved to differentiate the naevi from MMs successfully, with high specificity. Triple MITF/SOX10/SOX11 co-expression was seen in 9 out of 15 negative conventional pan-melanoma-cocktail cases. The independent prognostic value was proved for the conventional pan-melanoma cocktail (triple positivity for HMB-45, melan-A, and tyrosinase) and, independently for HMB-45 and tyrosinase, but not for melan-A, SOX10, or SOX11. As consequence, to differentiate MMs from benign naevi, melan-A should be substituted by SOX10 in the conventional cocktail. Although the conventional pan-melanoma cocktail, along with S100 can be used for the identification of melanocytic origin of tumor cells and predicting prognosis of MMs, the conventional-adapted cocktail (triple positivity for HMB-45, SOX10, and tyrosinase) has a slightly higher diagnostic specificity. SOX11 can be added to identify the aggressive MMs with risk for lymphatic dissemination and the presence of circulating tumor cells.

## 1. Introduction

Malignant melanoma (MM) is the twenty-first most frequently diagnosed malignant tumor worldwide, presenting almost a doubled mortality in the last two decades [1,2,3,4,5]. Due to its highly heterogeneous aspect, the histopathological diagnosis needs to be confirmed by immunohistochemical (IHC) markers. In daily practice, the IHC-panel usually includes S100 protein and at least one of the three members of the conventional pan-melanoma cocktail: Human melanoma black 45 (HMB45), MART1/melan-A, and tyrosinase [6,7,8]. In conventional pan-melanoma-cocktail-negative cases, it is recommended to add, for an appropriate differential diagnosis, other markers such as the microphthalmia transcription factor (MITF) [9] and the SRY-related high-mobility group box transcription factor 10 (SOX10) [10]. The proliferation grade is estimated based on the mitotic rate and the Ki67 percentage [11].

Except in the above-mentioned markers used for diagnosis, there are cases with uncertain differentiation in which the IHC panel needs to be enlarged. One of these antibodies is known as SOX11. It is a nuclear transcription factor belonging to the SOX-C proteins group, along with SOX4 and SOX12 [12,13,14].

This paper aimed to examine the possible role of SOX11 in the diagnosis and evolution of MM, in conjunction with clinicopathological factors, MITF, SOX10, and conventional pan-melanoma cocktail.

## 2. Materials and Methods

### 2.1. Selection Criteria

The study was done based on 105 consecutive patients with MM who underwent surgical excision between 2012 and 2018 (Table 1). Only patients who survived at least four months following surgery, with no neoadjuvant chemotherapy before excision, were included in this observational study. We did not examine cases of MMs in situ as well as cases with distant metastasis or those in whom follow-up data were not available. The patients’ follow-up ranged from 5 to 112 months, with a median time of 50.04 ± 4.89 months.

This retrospective study was conducted under the approval of the Ethical Committee of the “George Emil Palade” University of Medicine, Pharmacy, Sciences and Technologies of Targu Mures, Romania.

### 2.2. Histological Assessment and Tissue Microarray Construction

The first step was the histological re-evaluation of the MM cases. All the 105 MMs were re-staged based on the American Joint Committee on Cancer (AJCC) Cancer Staging Manual 8th edition [15] and the WHO Classification of Skin Tumors 4th edition [16]. Then, representative areas were marked, on Hematoxylin-Eosin (HE) sections, for the construction of the tissue microarray (TMA) blocks. Areas with necrosis, hemorrhage, or rich inflammatory infiltrate were avoided. The TMA blocks were generated from formalin-fixed paraffin-embedded tissue blocks (FFPE).

For the control group, to test the sensitivity and specificity of the examined markers, we included 44 cases of naevi. For both naevi and MMs, 10 cores-TMA blocks were constructed, using one tissue-core per case (4 mm diameter).

### 2.3. Immunohistochemistry—Technical Data

The IHC stains were performed to check the expression of the following antibodies: S100 protein (polyclonal; dilution 1:400; Dako, Agilent Technologies Inc., Glostrup, Denmark); Ki67 (clone MIB-1; dilution 1:100; Agilent, Santa Clara, CA, USA); SOX10 (clone A-2; dilution 1:100; Santa Cruz Biotechnology, Dallas, TX, USA); MITF (clone 34CA5; dilution 1:10; Leica Biosystems, Newcastle Ltd., Newcastle, UK); SOX11 (polyclonal; dilution 1:100; Sigma-Aldrich, St. Louis, MO, USA), and markers of the conventional pan-melanoma cocktail: HMB-45 (monoclonal; dilution 1:100; Cell Marque, Rocklin, CA, USA), melan-A (clone A103; dilution 1:100; Cell Marque, Rocklin, CA, USA), and tyrosinase (clone T311; ready to use (RTU); Leica Biosystems, Wetzlar, Germany).

The immunostains were performed for most of the markers (except for tyrosinase and MITF), using the semi-automated method and the detection system EnVisionTM FLEX (Agilent, Santa Clara, CA, USA). After deparaffinization and rehydration of the TMA sections, the activity of endogenous peroxidase was blocked by the EnVisionTM FLEX Peroxidase-Blocking reagent, for 10 min at room temperature. The antigen retrieval consisted of boiling in Tris/EDTA, pH 9 solution for 40 min at 95 ˚C using the PT Link 200 Pre-Treatment Module (Agilent, Santa Clara, CA, USA). Then, primary and secondary antibodies were incubated for 60 and 30 min, respectively, at room temperature (Dako EnVision™ FLEX/HRP detection reagent). The development was performed using the EnVision™ FLEX HRP Magenta Substrate Chromogen System (Agilent, Santa Clara, CA, USA) and the counterstain was done with Mayer’s Hematoxylin.

For tyrosinase and MITF, we used the BOND-MAX Fully Automated Immunostainer and the BOND Polymer Refine Red Detection kit (Leica Biosystems, Wetzlar, Germany) with ethylenediaminetetraacetic acid- (EDTA) based pH 9.0 epitope retrieval solution.

The negative control was assessed for each marker by omission of the primary antibody. For positive control, we used normal adipose tissue (for S100 protein), breast myoepithelial cells (for SOX10), solid pseudopapillary neoplasm of pancreas (for SOX11) [17], melanocytes from the epidermis (for HMB-45, MITF and tyrosinase), and Leydig cells–testis (for melan-A).

### 2.4. Interpretation of the IHC Stains

**Figure 1:**
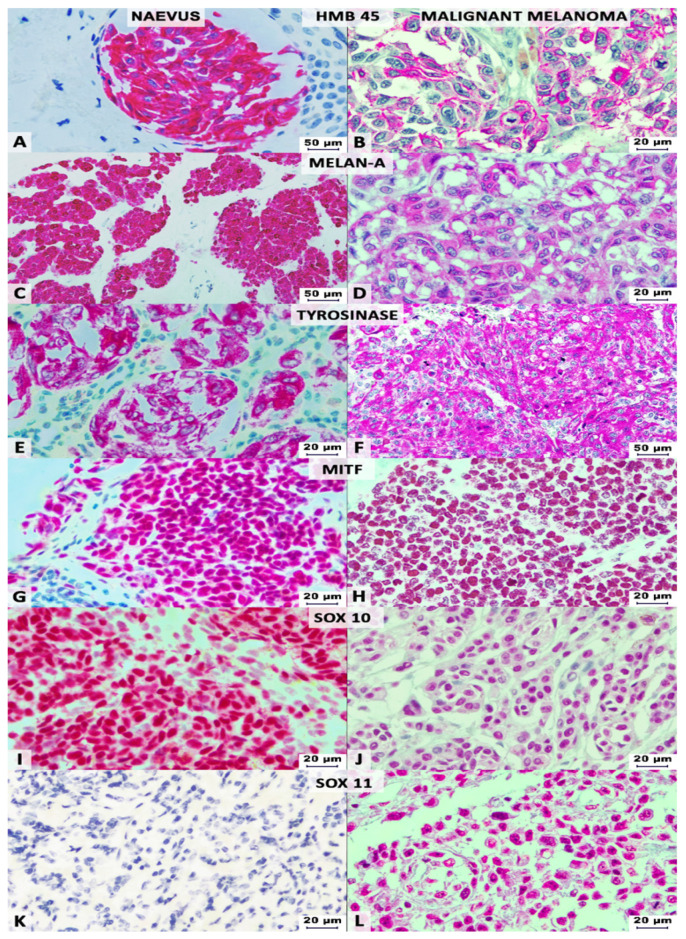
Immunohistochemical profile of naevi (left columns) vs. malignant melanomas (right columns), highlighted with Red Magenta Substrate Chromogen. HMB-45 can mark both naevi (**A**) and melanoma cells (**B**), same as melan-A (**C**,**D**), tyrosinase (**E**,**F**), microphthalmia transcription factor (MITF) (**G**,**H**) and SOX10 (**I**,**J**). No immunoreactivity is observed for SOX11 in naevi (**K**), but melanomas can present nuclear stain (**L**).

The immunoreactivity evaluation was performed in a blinded fashion by two senior pathologists (GS, JI) and one PhD student (BMA). For each of the examined markers, we used a cut-off value of 5% and counted the positive cells with obvious positivity, based on the percentage and intensity of the IHC stain. For SOX10, SOX11, MITF, and Ki67, we checked for tumors’ cells nuclear positivity, whereas the S100 protein and markers of the pan-melanoma cocktail were evaluated in the cytoplasm of the tumor cells (Figure 1).

The conventional pan-melanoma cocktail was considered positive if all the three IHC markers (HMB-45, melan-A, tyrosinase) were positive in the same lesion. The same rule was used for quantification of the conventional-adapted pan-melanoma cocktail, in which melan-A was replaced by SOX10. The proliferation index Ki67 was reported as a percentage of positive tumor cells vs. the total number of tumor cells, without considering the Ki67 positive-inflammatory cells.

### 2.5. Statistical Analysis and Survival Curves

Statistical analysis was done with the GraphPad Prism 8.4.3-software, free version (GraphPad Software, San Diego, CA, USA). Quantitative variables were evaluated for normality of distribution using the Kolmogorov–Smirnov test and were reported as mean and standard deviation (SD) while nominal variables were characterized by frequencies. To establish associations between clinicopathological factors, overall survival rate (OS), and IHC stains, we used the Chi-squared test and nonparametric Spearman test. Sensitivity and specificity were assessed for each marker, comparing the IHC expression in MMs vs. naevi, using the Wilson/Brown hybrid correction. Receiver-operating characteristic (ROC) curve analysis served to test the diagnostic power of the examined markers. The overall survival rate was estimated using the Kaplan–Meier curves and log-rank (Mantel–Cox) test. All the tests were two-tailed, and a *p*-value of <0.05 with a 95% confidence interval was considered statistically significant.

### 2.6. Gene Expression Levels, Survival Analysis, and Interactions in MMs, in Public Databases

Gene expression levels and survival analysis was performed using GEPIA (Gene Expression Profiling Interactive Analysis) program [18]. This online tool integrated data from the Cancer Genome Atlas (TCGA) and provides complex analysis related to gene expression and prognostic values related to key gene or gene signature, including those for Skin Cutaneous Melanoma (SKCM) [18].

Kaplan–Meier survival curves comparing high and low expression levels of SOX11, SOX10, and MITF in SKCM was done using GEPIA online interface. The miRNet database [19] was used for identification of mRNA–miRNA interaction.

## 3. Results

### 3.1. Naevi–Clinicopathological Factors

There were 35 benign and 9 dysplastic naevi which were diagnosed in patients with a median age of 33.36 ± 5.49 years (range 1–79 years) and a male to female ratio of 1:1.75 (16/28). They were localized on the trunk (*n* = 20; 45.45%), head and neck (*n* = 13; 29.55%), and limbs (*n* = 11; 25%).

### 3.2. MMs—Clinicopathological Factors and IHC Assessment

Examination of the 105 MMs, equally diagnosed in males and females, showed that almost one-third of them (*n* = 40; 38.09%) occurred in areas exposed to ultraviolet radiations (UVs): Head and neck skin (*n* = 17), the distal parts of superior and inferior limbs (*n* = 23). Most of the MMs were ulcerated nodular-type tumors, which were diagnosed in over 60% of the cases in advanced stages (pT3/pT4), with a high mitotic rate (Table 1).

Examination of the possible correlation between the IHC markers and clinicopathological parameters did not show a significant association for MITF or members of the conventional pan-melanoma cocktail with any of the examined parameters (Table 2).

The Ki67 index was positively correlated with Breslow thickness (r = 0.25; *p* = 0.009), presence of ulceration (r = 0.31; *p* = 0.001), mitotic rate (r = 0.34; *p* = 0.0004), and TNM stage (r = 0.3; *p* = 0.001) and related to the melanoma histological type (r = −0.28; *p* = 0.003).

**Figure 2:**
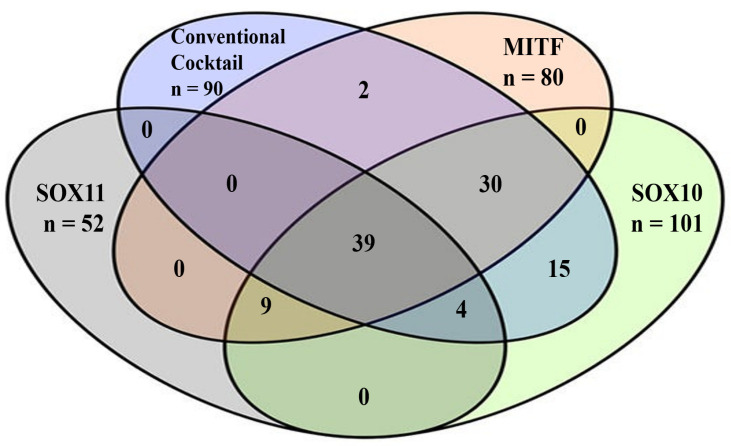
In skin cutaneous melanomas, the Venn diagram shows interaction between conventional pan-melanoma cocktail (triple positivity for HMB-45/melan-A/tyrosinase), SOX11, SOX10, and MITF.

SOX10 overexpression was found almost similar between superficial, nodular, and lentiginous type MM (Table 2). SOX10 expression was positively correlated with the conventional pan-melanoma cocktail (Figure 2).

SOX11 marked cases with lymphovascular invasion and 7 of the 9 MMs with neurotropism. A direct association was seen between SOX11 and Ki67 index (Table 2), as well as with MITF expression (Figure 2).

Double SOX10/SOX11 positivity was found to be directly correlated with lymphovascular invasion (r = 0.22; *p* = 0.07), as well as with MITF positivity (r = 0.37; *p* < 0.0001) and Ki67 index (r = 0.23; *p* = 0.017). Triple MITF/SOX10/SOX11 co-expression was directly correlated with Ki67 index (r = 0.24; *p* = 0.01). It was seen in 6 out of 9 cases with neurotropism.

### 3.3. IHC-Panel in MMs vs. Naevi

All the naevi cases proved positive for S100 protein and negative for SOX11. No IHC differences were seen between benign and dysplastic naevi. All the MMs were marked by S100 protein but, contrary to the naevi, SOX11 positivity was seen in almost half of the cases (49.52%).

In the group of naevi, the positivity rate for the conventional pan-melanoma cocktail was 43.18% (*n* = 19), respectively, 54.54% for HMB-45 (*n* = 24), 59.09% for tyrosinase (*n* = 26), and 84.09% for melan-A (*n* = 37), when examined individually. A similar rate of positivity was observed for SOX10 (*n* = 34; 77.27%) and MITF (*n* = 30; 68.18%). The rate of positivity for the conventional-adapted pan-melanoma cocktail (triple positivity for HMB-45, tyrosinase, and SOX10) was 38.63% (*n* = 17) (Table 3).

Most of the MM cases (85.71%) were positive for both conventional- and conventional-adapted pan-melanoma cocktail (triple positivity for HMB-45, tyrosinase, and SOX10) with a significantly higher rate compared with naevi (*p* < 0.0001). From the conventional pan-melanoma cocktail members, a significantly higher rate of positivity was highlighted by HMB-45 (92.38%) and tyrosinase (91.42%), but not by melan-A (92.38%) compared to their benign counterpart. The rate of positivity also proved significantly higher for SOX10 (96.19%), in MMs, compared with naevi (*p* = 0.0008), but did not differ (*p* = 0.31) for MITF (76.19%). The conventional-adapted cocktail presented a similar sensitivity, but a slightly higher specificity than the conventional cocktail (Table 3).

The Venn diagram outlined that 37.14% of the 105 MMs (*n* = 39) displayed positivity for all the examined markers: S100, SOX10, SOX11, MITF, and conventional pan-melanoma cocktail (Figure 2). A C-index of 0.7476 (standard error of 0.03469 and *p* < 0.0001), with a positive predictive value of 100% and a negative predictive value of 66.46%, was calculated for SOX11 showing its diagnostic utility.

**Figure 3:**
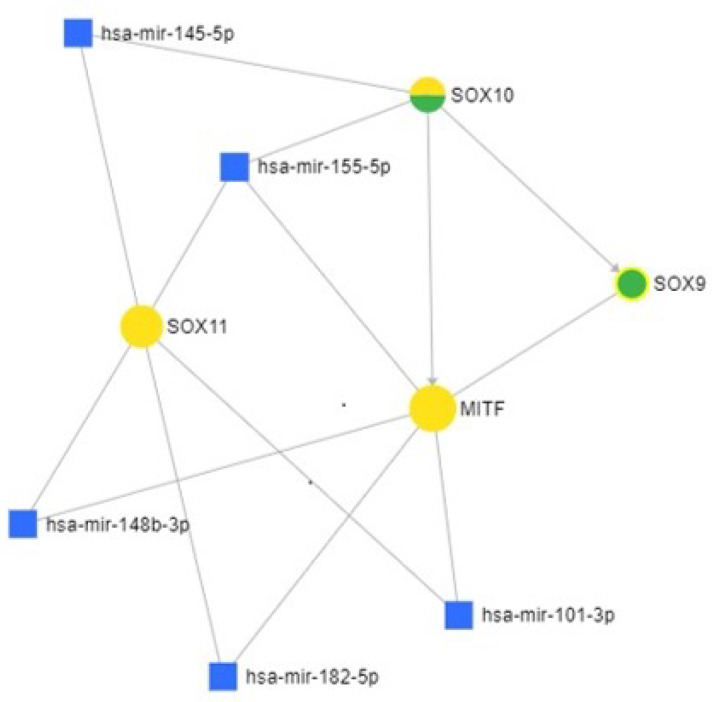
SOX11 SOX10, and MITF gene interaction generated using miRNET emphasizes direct interaction with miR-101-3p and miR-145-5p. Figure legend: Yellow circle: Gene; green circle: Transcription factor (TF); and blue square: miRNA.

The 15 negative conventional pan-melanoma-cocktail cases (Table 3) were represented by 9 cases with SOX11/SOX10/MITF triple positivity, 4 cases expressing only SOX10 positivity, and 2 cases that expressed HMB-45 and melan-A (Figure 2). The SOX11, SOX10, and MITF genes linkage is confirmed by the miRNet database (Figure 3). Their co-expression might serve as a diagnostic tool for pan-melanoma cocktail negative MMs. 

### 3.4. Survival Analysis

Complete follow-up data, after surgical excision, were available for all the 105 patients with MMs. The death event was directly correlated with age (r = 0.38; *p* < 0.0001), Breslow thickness (r = 0.49; *p* < 0.0001), ulceration (r = 0.21; *p* = 0.02), microsatellites (r = 0.21; *p* = 0.03), mitotic index (r = 0.39; *p* < 0.0001), Clark anatomic level (r = 0.28; *p* = 0.003), tumor diameter (r = 0.45; *p* < 0.0001), and the TNM stage (r = 0.38; *p* < 0.0001). A negative association of the histological type (r = −0.38; *p* = 0.001) and growth phase (r = −0.28; *p* = 0.004) with the OS was also revealed.

**Figure 4:**
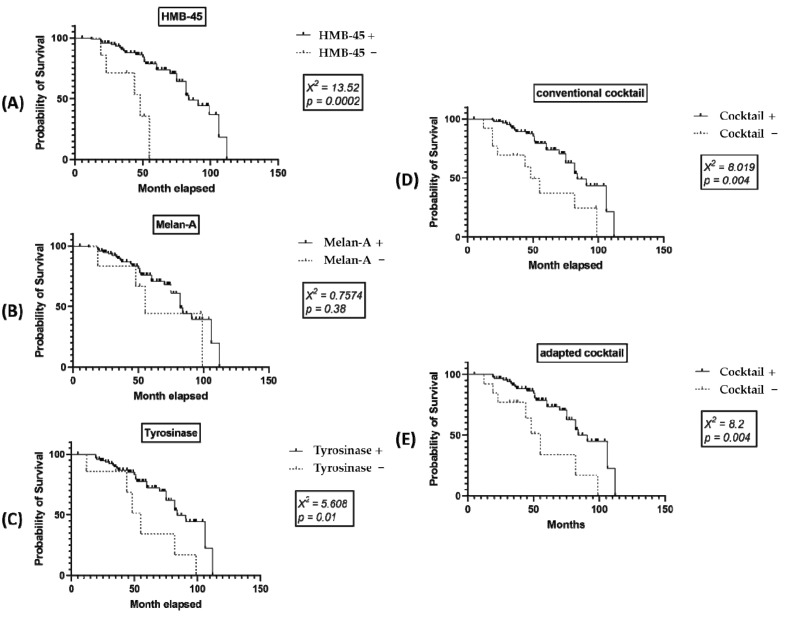
Univariate Kaplan–Meier survival analysis shows independent prognostic value for both (**D**) conventional pan-melanoma cocktail (triple positivity for HMB-45/melan-A/tyrosinase) and (**E**) conventional-adapted cocktail (triple positivity for HMB-45/SOX10/tyrosinase) and, independently, for (**A**) HMB-45 and (**C**) tyrosinase, but not for (**B**) melan-A.

Regarding the IHC markers, the conventional pan-melanoma cocktail, the conventional-adapted pan-melanoma cocktail and, particularly, HMB-45 and tyrosinase, but not melan-A, proved to have an independent prognostic value. A better OS rate showed patients with positive vs. negative cases (Figure 4).

**Figure 5:**
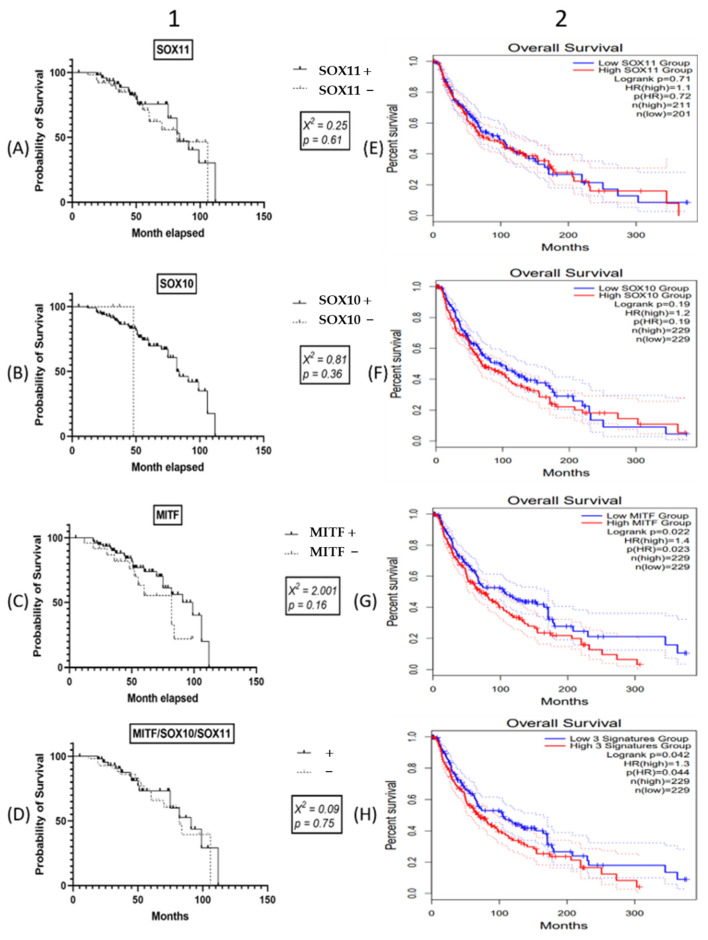
In the present cohort (**1**), univariate Kaplan–Meier survival analysis did not sustain the independent prognostic value of (**A**) SOX11, (**B**) SOX10, (**C**) MITF, or (**D**) co-expression of SOX11, SOX10, and MITF. In cutaneous melanomas, the TCGA dataset, which was examined using GEPIA (**2**), also inform the independent prognostic value of these factors (**E**) SOX11, (**F**) SOX10 and (**H**) co-expression of SOX11, SOX10, and MITF, except the (**G**) MITF gene signature.

In our material, any of SOX11, SOX10, MITF, or SOX11/SOX10/MITF co-expression did not present independent prognostic value for the 50 months-rate OS. This fact was confirmed by the TCGA datasets for SOX11, SOX10, and the SOX11/SOX10/MITF signature group, for long-term OS. TCGA showed an independent prognostic value for MITF gene signature if it is estimated for over 5 years. Patients with low MITF gene expression levels have a significantly longer OS (Figure 5).

## 4. Discussion

Our study showed that the S100 protein marks both naevi and MMs and does not have diagnostic utility for delineating the two lesions, same as melan-A and MITF, which revealed a low diagnostic sensitivity. As the two members of the pan-melanoma cocktail, HMB-45 and tyrosinase, proved to differentiate benign vs. malignant lesions, together with SOX10, the present paper suggests that, in the conventional cocktail, melan-A should be substituted by SOX10, for a better differentiation. However, melan-A remains useful for identification of melanocytic origin, in metastases. In difficult cases, SOX11 might be added to identify those aggressive MMs with neurotropism and risk for lymphovascular invasion.

In line with our results, some of the previously published studies showed that S100 and SOX10 remain the most sensitive markers for melanocytic tumors [6,10,20]. HMB-45, tyrosinase, and SOX10, alongside the Ki67 index, can aid in the distinction between benign and malignant melanocytic tumors [6,10]. SOX10 is also a sensitive marker for desmoplastic MMs [20].

As SOX10 correlates with the rate of lymph node metastasis [21], a double positivity of MMs for SOX10/SOX11 might be used as an indicator of the presence of tumor cells in lymphatic and systemic circulation. Once arrived in the metastatic organs, downregulation of SOX10 was reported in metastatic cells [21]. Triple SOX10/SOX11/MITF positivity might serve as a diagnostic tool for conventional pan-melanoma cocktail negative cases.

The abovementioned members of the SOX-E and SOX-C families, SOX10 and SOX11, interact from the embryogenesis period and seem to be involved in neural crest differentiation, respectively, neuro- and osteogenesis [3,12,13,14,22,23]. Their double positivity, in MMs, might be an indicator of melanomagenesis from the pluripotent neural crest and not from peripheral melanocytes. This might be the explanation of a higher risk for lymphatic dissemination of SOX10/SOX11 positive MMs. It is also known that, in adults, higher levels of SOX11 and SOX4 in the skin might induce tumorigenesis, because of reactivation of the embryonic transcriptional program [14,24].

Only one study, regarding the possible prognostic role of SOX11 in MMs, was previously conducted (2013) by Jian et al. [25], on a smaller group of patients (*n* = 40) suggesting that SOX11 may be used for differential diagnosis of benign vs. malignant melanocytic tumors. In MMs, the reported sensitivity (62.5%) [25] was slightly higher than that outlined by the present study (49.52%).

**Figure A1:**
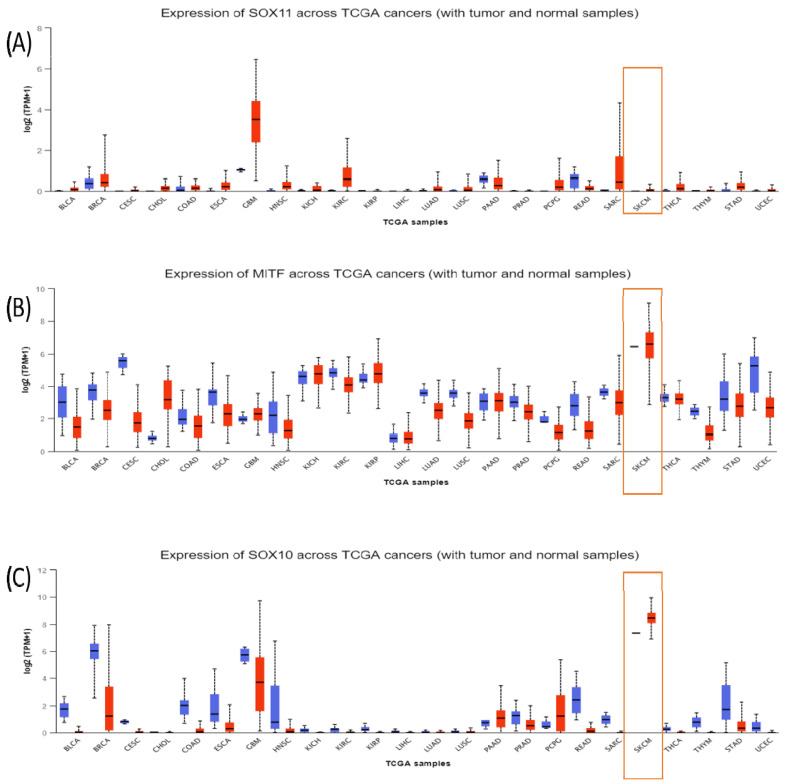
The mRNA expression profile across all tumor samples and paired normal tissue generate using Gene Expression Profiling Interactive Analysis project (GEPIA), (**A**) SOX11, (**B**) MITF, and (**C**) SOX10.

In other tumors, SOX11 is reported to play a role as an oncogene or, in contrast, a tumor suppressor gene, depending on tumor type [26,27] (Figure A1). It can exert an anti-apoptotic effect [26,28] or can block the tumor cell differentiation [26].

Except for MMs, overexpressed SOX11, as an indicator of aggressivity, was reported in mantle cell lymphoma [29,30], basal-like breast cancer [31], solid pseudopapillary neoplasm of the pancreas [17,32], and neuroendocrine tumors of the lung [33]. In these tumors, SOX11 might promote the epithelial-mesenchymal transition (EMT) [26,31,34] via the Wnt/β-catenin pathway [14,26]. Stimulation of angiogenesis, after interaction with pro-angiogenic markers such as platelet-derived growth factor A (PDGFA), was also outlined [26,29].

In MMs, it is still uncertain if β-catenin, as an indicator of the EMT, represses or promotes melanoma invasion [34]. It is also unknown if the EMT is mediated via the Wnt/β-catenin or the Wnt/SOX4 pathway [26].

As Jian et al. showed an association between SOX11 and lymph node metastasis [25] and our results proved its association with the lymphovascular invasion rate, before the occurrence of metastasis, it is tempting to believe that SOX11 can be used for identification of those MMs with high risk of presence of circulating cells before clinical diagnosis of lymph node metastases. Based on this fact, large cohorts need to prove the possible role of SOX11 for identification and targeted therapy of such MMs.

The limitations of the study consist in the relatively small number of examined cases, no inclusion of metastatic cases, and use of TMA blocks. The TMA sections might affect interpretation of the intratumorally heterogeneity. Large cores, long-term follow-up, and the complex IHC panel compensated these limitations. Contrary to Jian et al., who published the only study regarding the role of SOX11 in MMs, our findings did not outline any relationship between SOX11 expression and histologic type, tumor location, or UV-exposure [25]. This difference between our cohort and those of Jian et al. [25] was probably because most of our patients showed nodular tumors, whilst previously reported data comprised only superficial spreading and lentiginous types of MM.

## 5. Conclusions

In daily practice, the conventional pan-melanoma cocktail (HMB-45, melan-A, and tyrosinase), along with S100 can be successfully used for the identification of the melanocytic origin of tumor cells. Differentiation from benign naevi and MMs should be done based on a conventional-modified cocktail in which melan-A is replaced by SOX10. SOX11 can be added to identify the aggressive MMs with risk for lymphatic dissemination and the presence of circulating tumor cells. If SOX10/SOX11 double positivity might serve as an indicator of lymphatic invasion, triple SOX11/SOX10/MITF gene interaction might induce genesis of a subtype of MMs which do not express positivity for the conventional pan-melanoma cocktail.

## Figures and Tables

**Table 1:** Clinicopathological parameters of patients with malignant melanoma.

Variable	N = 105 (%)
Age (years)	63.63 ± 14.48 (range 30–90)
Gender: Male: Female	52:53 (1:1.01)
Histological type	
Nodular	76 (72.37)
Superficial	18 (17.15)
Lentiginous	11 (10.48)
Thickness (Breslow)	(Median: 6.73 ± 8.25, range 0.4–60)
≤1 mm	20 (19.05)
>1 to ≤2 mm	15 (14.29)
>2 to ≤4 mm	14 (13.33)
>4 mm	56 (53.33)
Ulceration	74 (70.47)
Microsatellites	19 (18.09)
Mitotic Rate (mm^2^)	(Median: 10.09 ± 11.9, range 0–57)
<5	42 (40)
≥5	63 (60)
TILs (Tumor-Infiltrating Lymphocytes)	
Not identified	30 (28.58)
Brisk	19 (18.09)
Non-Brisk	56 (53.33)
Lymphovascular Invasion	23 (21.9)
Neurotropism	9 (8.57)
Tumor regression	36 (34.28)
Anatomic Level (Clark)	
I	1 (0.95)
II	11 (10.48)
III	13 (12.38)
IV	58 (55.24)
V	22 (20.95)
Tumor location	
Anterior Trunk	18 (17.15)
Posterior Trunk	29 (27.61)
Head and Neck	17 (16.19)
Superior Limb	16 (15.24)
Inferior Limb	25 (23.81)
Tumor satellites	12 (11.42)
Tumor size (mm)	23.45 ± 15.78 (range 6–110)
Tumor stage	
pT1	20 (19.05)
pT2	17 (16.19)
pT3	14 (13.33)
pT4	54 (51.43)
Deep Margin distance (mm)	8.41 ± 5.21 (range 6–22.8)
Peripheral Margins distance (mm)	7.67 ± 5.9 (range 1–35)
Growth Phase	
Radial	20 (19.05)
Vertical	85 (80.95)

**Table 2:** Association of immunohistochemical markers with clinicopathological parameters.

Parameters	N	SOX11				SOX10				MITF				Conventional Cocktail			
		+	−	R	*p*	+	−	r	*p*	+	−	r	*p*	+	−	R	*p*
Gender																	
Male	52	29	23	0.12	0.2	51	1	0.09	0.32	39	13	0.09	0.64	45	7	0.02	0.81
Female	53	23	30			50	3			41	12			45	8		
Age (years)																	
≤60	39	18	21	0.003	0.97	37	2	0.1	0.26	31	8	−0.03	0.89	35	4	−0.1	0.33
>60	66	34	32			64	2			49	17			55	11		
Histologic type																	
Nodular	76	41	35	−0.13	0.17	75	1	−0.24	0.01	58	18	0.15	0.47	64	12	0.07	0.49
Superficial	18	7	11			15	3			14	4			16	2		
Lentiginous	11	4	7			11	0			8	3			10	1		
Thickness (Breslow)																	
≤1 mm	20	5	15	0.14	0.15	19	1	0.11	0.26	14	6	−0.08	0.7	18	2	−0.17	0.08
>1 to ≤2 mm	15	9	6			14	1			14	1			14	1		
>2 to ≤4 mm	14	8	6			13	1			10	4			13	1		
>4 mm	56	30	26			55	1			42	14			45	11		
Ulceration																	
Present	74	40	34	0.13	0.17	72	2	0.09	0.37	59	15	−0.01	0.96	62	12	−0.09	0.37
Absent	31	12	19			29	2			21	10			28	3		
Microsatellites																	
Present	19	12	7	0.13	0.18	18	1	−0.03	0.72	16	3	0.18	0.37	14	5	−0.16	0.1
Absent	86	40	46			83	3			64	22			76	10		
Mitotic Rate (mm^2^)																	
<5	42	19	23	0.1	0.3	39	3	0.14	0.17	33	9	0.1	0.6	37	5	−0.06	0.56
≥5	63	33	30			62	1			47	16			53	10		
TILs																	
Not identified	30	17	13	−0.07	0.44	30	0	−0.12	0.2	24	6	0.25	0.2	26	4	−0.08	0.44
Brisk	19	10	9			19	0			18	1			18	1		
Non-Brisk	56	25	31			52	4			38	18			46	10		
Lymphovascular Invasion																	
Present	23	16	7	0.21	0.02	23	0	0.1	0.28	19	4	−0.06	0.77	21	2	0.09	0.38
Absent	82	36	46			78	4			61	21			69	13		
Neurotropism																	
Present	9	7	2	0.17	0.07	9	0	0.06	0.53	7	2	−0.02	0.92	8	1	0.03	0.77
Absent	96	45	51			92	4			73	23			82	14		
Tumor regression																	
Present	36	17	19	−0.02	0.79	34	2	−0.06	0.51	27	9	−0.15	0.44	31	5	0.01	0.91
Absent	69	35	34			67	2			53	16			59	10		
Anatomic Level (Clark)																	
I-III	25	10	15	0.1	0.29	23	2	0.06	0.5	19	6	−0.11	0.58	22	3	0.01	0.92
IV-V	80	42	38			78	2			61	19			68	12		
UV exposure																	
Present	40	20	20	0.01	0.87	38	2	−0.04	0.63	30	10	−0.21	0.28	32	8	−0.13	0.2
Absent	65	32	33			63	2			50	15			58	7		
TNM stage																	
pT1-pT2	37	15	22	0.13	0.18	35	2	0.09	0.32	29	8	0.01	0.96	34	3	−0.14	0.15
pT3-pT4	68	37	31			66	2			51	17			56	12		
Ki67 Index																	
≤10	74	31	43	0.23	0.01	70	4	0.17	0.07	53	21	0.33	0.0005	64	10	0.34	0.72
>10	31	21	10			31	0			27	4			26	5		

**Table 3:** Sensitivity and specificity of immunohistochemical markers in melanomas vs. naevi.

IHC Marker	Melanoma		Naevi		Sensitivity	Specificity	
	+	−	+	−	%	%	*p* Value
S100	105	0	44	0	100%	0%	0.99
HMB-45	97	8	24	20	92.38%	45.45%	<0.0001
Melan-A	97	8	37	7	92.38%	15.91%	0.14
Tyrosinase	96	9	26	18	91.43%	40.91%	<0.0001
MITF	80	25	30	14	76.19%	31.82%	0.31
SOX10	101	4	34	10	96.19%	22.73%	0.0008
SOX11	52	53	0	44	49.52%	100%	<0.0001
Conventional cocktail ^1^	90	15	19	25	85.71%	56.82%	<0.0001
Conventional-adapted cocktail ^2^	90	15	17	27	85.71%	61.36%	<0.0001

^1^ Triple positivity for HMB-45/melan-A/tyrosinase. ^2^ Triple positivity for HMB-45/SOX10/tyrosinase.

## Appendix A

To further reveal the transcriptional levels of SOX11, SOX10, and MITF in SKCM, we next conducted the analysis in Gene Expression Profiling Interactive Analysis (GEPIA) (Figure A1).
